# Effect of Addition of Rubber Granulate and Type of Modified Binder on the Viscoelastic Properties of Stone Mastic Asphalt Reducing Tire/Road Noise (SMA LA)

**DOI:** 10.3390/ma13163446

**Published:** 2020-08-05

**Authors:** Wladyslaw Gardziejczyk, Andrzej Plewa, Raman Pakholak

**Affiliations:** Faculty of Civil Engineering and Environmental Sciences, Bialystok University of Technology; 15-351 Bialystok, Poland; a.plewa@pb.edu.pl (A.P.); r.pakholak@doktoranci.pb.edu.pl (R.P.)

**Keywords:** asphalt mixtures, stone mastic asphalt, modified binder, crumb rubber, rubber granulate, hysteresis loop, stiffness modulus

## Abstract

The use of rubber granulate in the composition of asphalt mixtures, as well as the use of poroelastic layers, is indicated by many research centers as a factor with a positive effect on tire/road noise reduction. Attention is however paid to their lower structural durability compared to asphalt concrete (AC) or stone mastic asphalt (SMA). Stone mastic asphalt reducing tire/road noise (SMA LA) layers have also been recently used as low-noise road surfaces. The article presents the test results of viscoelastic properties of asphalt mixtures SMA8 LA, SMA8 LA containing 10%, 20%, and 30% of rubber granulate, with bitumen 50/70, bitumen 50/70 modified with styrene butadiene styrene (SBS) copolymer, crumb rubber, and mixtures with bitumen modified simultaneously with crumb rubber and SBS copolymer. The reference asphalt mixture was the porous asphalt (PA8). The presented results of water damage resistance, degradation resistance in the Cantabro abrasion loss test, stiffness modulus as a function of temperature and hysteresis loop proved that the amount of rubber granulate and the type of binder significantly affect the values of these parameters. Attention was paid to the possibility of using the results of uniaxial cyclic compression tests when determining the proportion of rubber granulate in SMA8 LA mixtures. Tests of hysteresis loops and stiffness modulus confirm much higher elasticity of SMA8 LA mixtures with rubber granulate as compared to mixtures without the addition of granulate.

## 1. Introduction

The construction of low-noise road surfaces or the so-called “quiet” road surfaces is a beneficial and effective way of combating excessive noise levels from road traffic [1,2,3,4,5]. However, the opinions on the acoustic efficiency of porous asphalt surfaces over a longer period of operation are inconclusive [6]. This is mainly due to their lower structural durability, higher construction costs and difficulties in winter maintenance. As a result, thin asphalt layers are more and more often used as a solution reducing the noise from road traffic.

In Germany, a stone mastic asphalt with increased air void content of the SMA LA type was developed [7]. Two types of this mixture were proposed: SMA5 LA (layer thickness 2.0–3.0 cm) and SMA8 LA (layer thickness 2.5–4.0 cm). The air void content was determined in the range from 9% to 14%. The noise reduction of SMA LA layers compared to traditional solutions is from 2.5 dB to 4.0 dB according to the CPX (Close Proximity) method.

The issue of the effect of rubber granulate in asphalt mixtures on the reduction of tire/road noise levels has been widely analyzed in scientific and research centers [8,9,10]. Granulate can be added in the “wet process” (as a modification of bitumen) or in the “dry process”—partially replacing mineral aggregate. Studies have shown that the rubber addition in the asphalt mixture in conjunction with the optimal air void content can significantly improve its acoustic properties. It is an essential component of poroelastic layers and accounts for at least 20% of the bitumen-rubber-aggregate mixture.

As part of the research in the PERSUADE (Poro-Elastic Road Surfaces for the Advanced Defense of the Environment) project [11,12] it was found, among others that considerably smaller rigidity compared to traditional solutions and a greater air void content determine the reduction of noise level in such layers. However, the structural durability of the poroelastic road surfaces produced so far was definitely lower than assumed. Further works on problems related to the construction of poroelastic layers are underway. This applies to the parameters characterizing both modified bitumen binders as well as bitumen-rubber-aggregate mixture.

“Good bitumen” is a binder with high rigidity at high operating temperatures occurring in summer and adequate flexibility during exposure to negative temperatures [13,14,15]. The analysis presented in [16] proves that binders that can be used for poroelastic road surfaces should have a Fraass Breaking Point within −20 °C and a softening point above 65 °C, with a viscoelastic range approximately equal to 85 °C.

The use of multi-scale performance tests to determine the optimal amount of crumb rubber in SMA mixtures as a material that partially replaces the filler and sand fraction, forming with asphalt mastic and mortar, respectively, is discussed in [17]. The effects of using New Dry-Hybrid Technology have been investigated from the mastic, mortar, and mixture points of view. The technical properties of the mastic and mortar were determined in dynamic shear and torsion tests, and then the test results were compared to the tests of the stiffness modulus of SMA mixtures determined in the direct tension-compression test (DTC Stiffness Modulus Test). The authors showed that the rheological properties of the mastic are affected by the presence of crumb rubber. This means that the stiffness of bitumen and the thermal sensitivity are related to the rheo-mechanical properties of the mastic and mortar. It was found that the addition of crumb rubber allows for more durable and environmentally friendly pavements.

Viscoelastic properties (e.g., stiffness modulus) of asphalt mixtures are significantly affected not only by the temperature and time of exposure to the load, but also by the type and amount of binder used [18,19,20]. It has been established that dynamic tests are good methods for determining the rheological parameters of materials designed for elastic surfaces. For bitumen-rubber-aggregate mixtures, more detailed tests should include tests on the stiffness modulus as a function of temperature, modified cyclic uniaxial compression test (determination of the hysteresis loop), water damage resistance, and resistance in the Cantabro abrasion loss test.

In the study of poroelastic mixtures presented in [21], two procedures were used: stress-strain tensile test and dynamic modulus test. Based on the results of stress and strain changes, their elastic properties were determined. Hysteresis loops between stress change and strain were plotted. Test results showed that all samples from poroelastic mixtures were stable and resistant to higher hysteresis losses during many subsequent testing cycles. It was found that along with the increase in the content of rubber granulate there were smaller losses determined when analyzing the area of the hysteresis loop. Dynamic stiffness modulus for poroelastic mixtures changed depending on the proportions between mineral aggregate and rubber granulate. Mixtures with higher aggregate content and lower rubber content had higher stiffness modulus. Comparing stiffness modulus of poroelastic mixtures with typical asphalt concrete confirmed that poroelastic mixtures are characterized by higher elasticity by about 20–1500 times. This means that poroelastic mixtures have better noise reduction capacity than car tires.

It has been shown in [22,23] that for the assessment of the rheological properties of asphalt mixtures, a good solution is to perform dynamic stiffness modulus (E*) tests in the DTC-CY (Dynamic Test Compression on cylindrical specimens). The tests were carried out at varying loads and at different temperatures. The stiffness of the analyzed mixtures was also assessed by testing the stiffness modulus in IT-CY (Indirect Tensile Test on cylindrical specimens) at various test temperatures.

As part of the SEPOR (Safe Eco-friendly Poroelastic Road Surface) project [24,25] research is carried out on poroelastic mixtures with a significant content of rubber granulate. This article presents the results of testing the effects of modification of bitumen 50/70 and the effect of the addition of rubber granulate in the “dry process” to stone mastic asphalt reducing tire/road noise (SMA8 LA) on their viscoelastic properties.

## 2. Materials

In the first stage, the following types of bitumen binders were tested: bitumen 50/70 (the reference binder), bitumen 50/70 modified with 5% SBS copolymer (SBSM-5), bitumen 50/70 modified with 10% crumb rubber (CRM-10) and bitumen 50/70 modified with 10% crumb rubber and 2% SBS copolymer (SBSM-2 + CRM-10). A copolymer—Kraton 1192 with 30% content of styrene was used for the binder modification; its molecular weight is 1.38 × 10^5^ g/mol. The crumb rubber with grain size of 0/0.8 mm used for bitumen modification came from the recycling of used car tires. The modification process consisted of heating bitumen 50/70 to 180 ± 5 °C, then adding the appropriate number of modifiers: 5% SBS copolymer or 10% crumb rubber, or 2% SBS copolymer and 10% crumb rubber at the same time.

A mechanical agitator rotating at a speed of 700 rpm, made at the Bialystok University of Technology (Bialystok, Poland), was used to homogenize the additives in the bitumen. The effective mixing time was 1 h.

Tests of bitumen binders before and after technological aging process included:

- penetration at temperatures: 5 °C, 15 °C and 25 °C;
- softening point with ring and ball method (R & B);
- dynamic viscosity at 90 °C, 110 °C and 135 °C;
- strain energy along with the determination of the maximum tensile force at temperatures of 5 °C, 15 °C and 25 °C;
- elastic recovery at temperatures of 15 °C and 25 °C.

Simulation of technological aging process in laboratory conditions was performed by rolling thin film oven test (RTFOT) method according to the relevant standard [26]. The tested technical properties of modified binders are presented in Table 1.

Amphibolite aggregate was used for the production of asphalt mixtures and bitumen-rubber-aggregate mixtures. The physical properties of the aggregate used are summarized in Table 2.

In the tested mixtures, rubber granulate with a grain size of 1/4 mm was added in the “dry process” replacing 10%, 20% and 30% of mineral aggregate by volume, improving the elasticity of mixture. Rubber granulate was heated at 60 °C for 30 min and then added directly to the mixer during the preparation of the asphalt mixtures.

**Figure 1:**
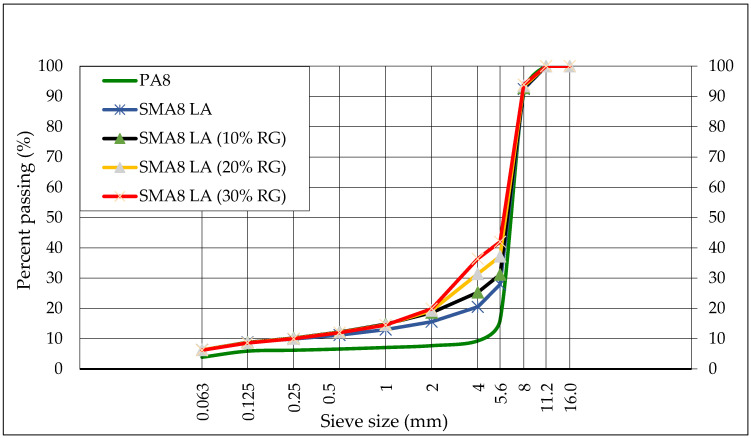
The particle size distribution of tested mixtures.

The following mixtures were tested: stone mastic asphalt SMA8 LA and SMA8 LA with 10%, 20% and 30% rubber granulate. Porous asphalt PA8 was used as the reference mixture. Test specimens were prepared in the Marshall compactor in accordance with standard [27] (mold diameter 101.6 mm; compaction: 50 blows on each side to a height of 63.5 ± 2.5 mm). The particle size distribution is shown in Figure 1. The binder content, air void content and asphalt mixtures bulk density are shown in Table 3.

## 3. Methods and Experimental Results

### 3.1. Water Damage Resistance

Water damage resistance test ITSR (Indirect Tensile Strength Ratio), performed according to technical requirements [28], provides for the division of specimens into a dry set and a wet set. Specimens were prepared in the Marshall compactor, 35 blows per side. Specimens from the dry set were stored at room temperature at 25 ± 5 °C. The wet set specimens in the first stage were soaked with water, then for 72 h they were conditioned in a water bath at 40 °C. In the next stage, the specimens saturated with water were frozen at −18 °C, and then again conditioned in water at 25 °C for 24 h. In the third stage, the unconditioned and conditioned specimens were subjected to compressive strength test ITS (Indirect Tensile Strength) in accordance with the relevant standard [29] (dry set—ITS_d_, wet set—ITS_w_). The ITSR water damage resistance was determined on the basis of Equation (1). The results of the ITSR examinations are summarized in Table 4.

(1) $$\mathrm{ITSR}=\frac{{\mathrm{ITS}}_{w}}{{\mathrm{ITS}}_{d}}\times 100\%$$

### 3.2. Cantabro Abrasion Loss Test

To assess the effect of bitumen binder on the resistance to degradation of asphalt and bitumen-rubber-aggregate mixtures, a Cantabro test was carried out in accordance with the relevant standard [30] The author of the article [31] considers it appropriate to perform the Cantabro test not only for porous asphalt mixtures. This approach was also applied in the present study. Specimens for the Cantabro abrasion loss test were prepared in the Marshall compactor (50 blows per side). Specimens with an initial mass P_1_ of the sample at 25 °C were placed in a Los Angeles abrasion test drum (without metal balls). After 300 drum revolutions at a constant speed of 30–33 rpm, the specimen mass P_2_ was again determined. The degradation of mixtures was calculated on the basis of Equation (2) and the test results are given in Table 4.

(2) $$P=\frac{P_{1}-P_{2}}{P_{1}}\times 100\%$$

### 3.3. Stiffness Modulus According to the Indirect Tensile Method (IT-CY)

The determination of stiffness modulus IT-CY was performed on cylindrical specimens with a diameter of d = 101.6 mm and height h = 63.5 ± 2.5 mm, in accordance with the relevant standard [32]. The tests were carried out at temperatures: 5 °C, 15 °C and 25 °C. In the first phase, the specimens were subjected to the initial loads—10 initial impulses enabling the determination of the appropriate force value and duration of the loading to achieve a specific horizontal deformation of the specimen. The raise time to its maximum value must be 124 ± 4 ms. The maximum value of the loading force is determined in such a way as to obtain the peak value of the instant horizontal deformation equal to 0.005% of the specimen diameter. The impulse repetition time is 3.1 ± 0.1 s. The second phase of the test is the next five impulses, referred to as the proper determination. The test result is the average value of five specimen load impulses, noting the variability of the applied force (F) and the level of specimen deformation in relation to its diameter (z). The examination for each individual specimen was made in two planes perpendicular to each other. The stiffness modulus IT-CY was calculated based on the formula (3):

(3) $$S_{m}=\frac{F\times \left(\nu +0.27\right)}{z\times h}$$

The test results of stiffness modulus for SMA LA and PA mixtures are presented in Table 5.

### 3.4. Modified Uniaxial Cyclic Compression Test

**Figure 2:**
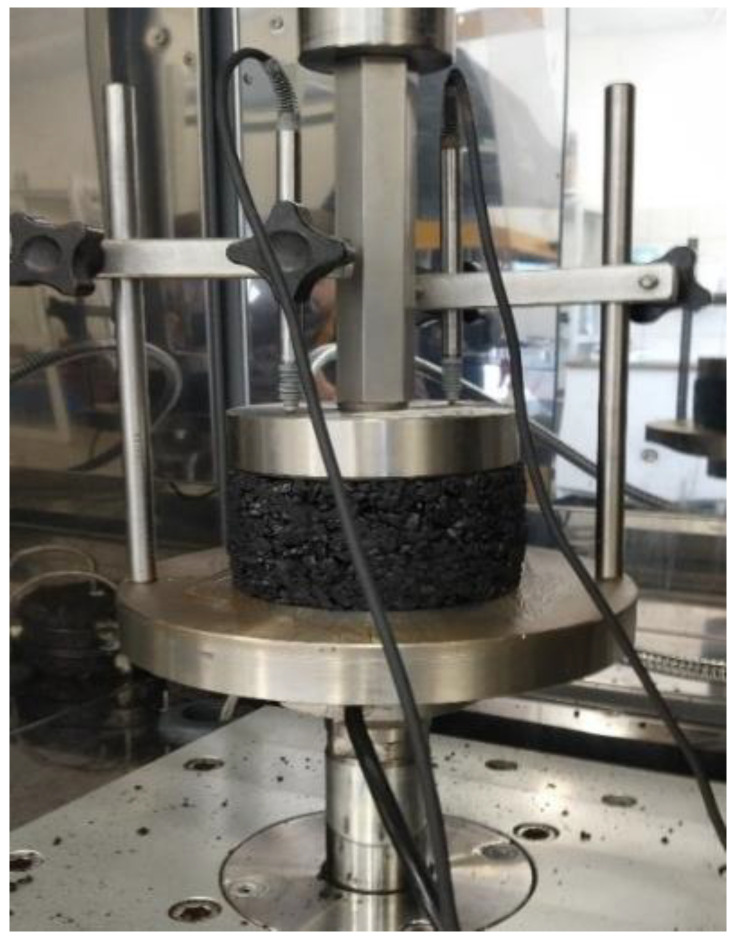
Test stand for the determination of uniaxial cyclic compression.

The modified method of uniaxial cyclic compression test in accordance with standard [33] was used to assess the elastic properties of mixtures from SMA8 LA and PA8 containing various modified binders. The research process provides for calculation the axial deformation of cylindrical specimens at repetitive loading. Specimens for the test were prepared in the Marshall compactor (50 blows on each side, specimen diameter 101.6 mm, height 60 ± 2 mm). The tests were carried out at 15 °C, 25 °C and 35 °C. The specimens were placed between two parallel plates, the upper one with a diameter of 100 mm, the lower one—185 mm. The test stand for the determination of uniaxial cyclic compression (Manufacturer: Pavetest Pty.Ltd, Endeavour Hills, Victoria, Australia) is shown in Figure 2.

In the first stage of the test, the specimens were preloaded with 100 kPa during 600 s. In the second stage, the specimens were subjected to the pulse loads with the pulse duration of 500 ms. The duration of the relaxation between the pulses was also 500 ms. The values of the loading stress were 850 kPa. After 300 cycles of pulse loading and relaxation, hysteresis loops were determined based on the results obtained from the stress/deformation relationship.

Elastic hysteresis [34] is one of the most important relationships describing the relaxation processes of materials, the essence of which is to delay the “development” of deformation of matter (including viscoelastic materials) under load. The hysteresis loop describes the energy losses resulting from internal friction occurring in the materials. The effect of elastic hysteresis depends on the loading speed and test temperature. The energy "accumulated" in the material is determined as the area of the hysteresis loop which is the difference in the work used to load the material (Equation (4)) and its relaxation (Equation (5)) after unloading:

(4) $$A_{\mathrm{loading}}=\overset{E_{2}}{\underset{o}{\int }}\sigma_{1}\mathrm{dE}$$

(5) $$A_{\mathrm{relaxation}}=\overset{E_{1}}{\underset{E_{2}}{\int }}\sigma_{2}\mathrm{dE},$$

where $\sigma_{1}$ and $\sigma_{2}$ stress change function during loading and relaxation.

The area of the hysteresis loop was determined from the Equation:

(6) $$S=A_{\mathrm{loading}}-A_{\mathrm{relaxation}}=\overset{E_{2}}{\underset{o}{\int }}\sigma_{1}\mathrm{dE}-\overset{E_{1}}{\underset{E_{2}}{\int }}\sigma_{2}\mathrm{dE}$$

The results of the area of the elastic hysteresis loop of the SMA8 LA and PA8 mixtures, determined according to the formula 6, are presented in Table 5.

## 4. Analysis of Results and Discussion

### 4.1. Water Damage Resistance ITSR

**Figure 3:**
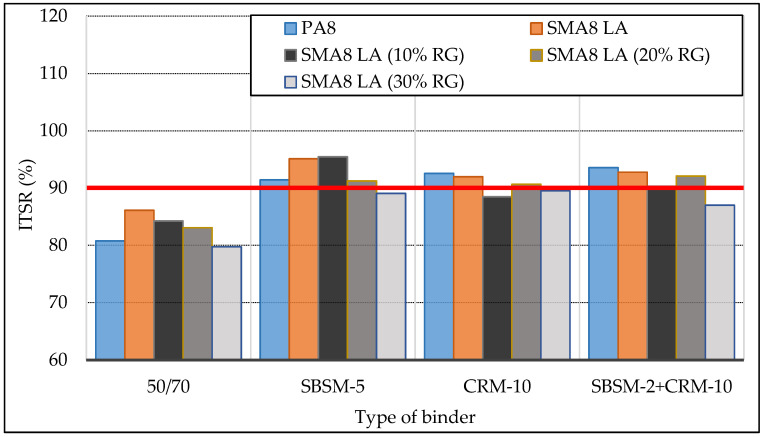
Water damage resistance ITSR.

Figure 3 shows the results of tests on the sensitivity of SMA8 LA and PA8 mixtures to water damage resistance ITSR.

The results of the ITSR index of asphalt mixtures confirm that the type of binder used affects their water damage resistance. Asphalt mixtures with bitumen 50/70 do not meet the requirements for water damage resistance according to TR-2 (ITSR ≥ 90%). SMA8 LA and PA8 mixtures with modified bitumen’s meet these requirements in most cases. Modified binders increase the ITSR index of the tested asphalt mixtures by about 10% compared to the mixtures with bitumen 50/70. Mixtures SMA8 LA (30% RG) and SMA8 LA (10% RG) with the bitumen-rubber binder (CRM-10) obtained slightly lower ITSR values than the TR-2 requirements. The highest water damage resistance was achieved by SMA8 LA (10% RG) and SMA8 LA mixtures with the copolymer modified bitumen SBSM-5, the mixture of porous asphalt (PA8) with bitumen modified with copolymer and crumb rubber (SBSM-2+CRM-10).

Analysis of the test results showed that the increase in the replacement of the mineral aggregate by rubber granulate reduces the water damage resistance of mixtures. The reason for this may be the fact that the rubber granulate is characterized by greater roughness of the grain surface. Increasing the amount of bitumen would probably improve the water damage resistance (ITSR) of asphalt mixtures with rubber granulate. However, this requires more detailed research.

### 4.2. Cantabro Abrasion Loss Test

**Figure 4:**
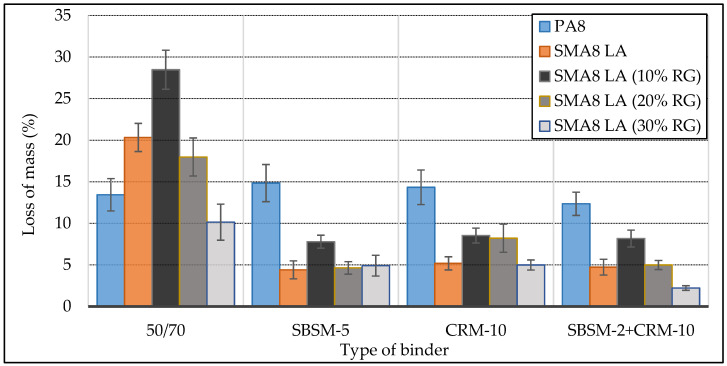
Cantabro test results.

Figure 4 shows the results of tests on the resistance of SMA8 LA and PA8 mixtures to degradation determined in the Cantabro abrasion loss test.

Based on the tests results on the resistance of SMA8 LA mixtures to degradation, it was found that the increase in the addition of rubber granulate improves their resistance to degradation. This fact is particularly visible for mixtures SMA8 LA with bitumen 50/70. Mixtures SMA8 LA (30% RG) with copolymer modified bitumen and crumb rubber (SBSM-2 + CRM-10) achieved the highest degradation resistance. The mixtures SMA8 LA with modified binders are characterized by much greater resistance compared to SMA8 LA with bitumen 50/70. The application of the modified binders improves the resistance of SMA mixtures to degradation by 3–4 times. However, the type of modified binder practically does not affect the degradation of SMA8 LA mixtures. The highest susceptibility to degradation among SMA8 LA mixtures achieved SMA8 LA (10% RG)—28.5%.

The tests also prove that the type of bitumen binder practically does not affect the results of the determination of porous asphalt PA8 mixtures (determined values from 12.35% to 14.48%).

### 4.3. Stiffness Modulus (IT-CY)

**Figure 5:**
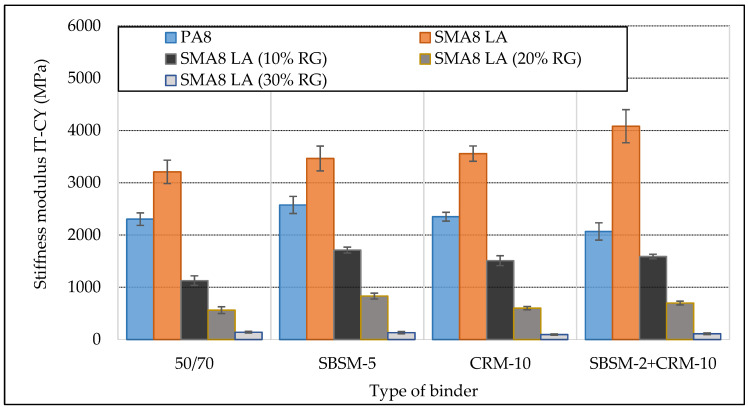
Stiffness modulus IT-CY of SMA LA8 and PA8 mixtures at 15 °C.

Figure 5 shows the results of tests on the stiffness modulus IT-CY of SMA8 LA and PA8 mixtures at 15 °C. This is the equivalent temperature of layers from asphalt mixtures under climatic conditions in Poland.

The test results on the stiffness modulus IT-CY of SMA8 LA mixtures confirmed the fact that the increase in the replacement of mineral aggregate by rubber granulate reduces the value of stiffness modulus. Mixtures SMA8 LA, SMA8 LA (10% RG) and SMA8 LA (20% RG) with modified binders have higher stiffness modulus values compared to the same mixtures with bitumen 50/70. The mixture SMA8 LA (30% RG) with unmodified bitumen 50/70 showed slightly higher modulus values compared to mixtures with modified bitumen. The highest values of stiffness modulus achieved the mixture SMA8 LA with modified bitumen with copolymer and crumb rubber (SBSM-2 + CRM-10)—4082 MPa. This is a 25% increase in modulus value compared to the same mixture with bitumen 50/70. The amount of added rubber granulate has a significant impact on the value of the stiffness modulus in SMA mixtures. An increase in the amount of rubber by 10% reduces the value of stiffness modulus on average 2–3 times.

**Figure 6:**
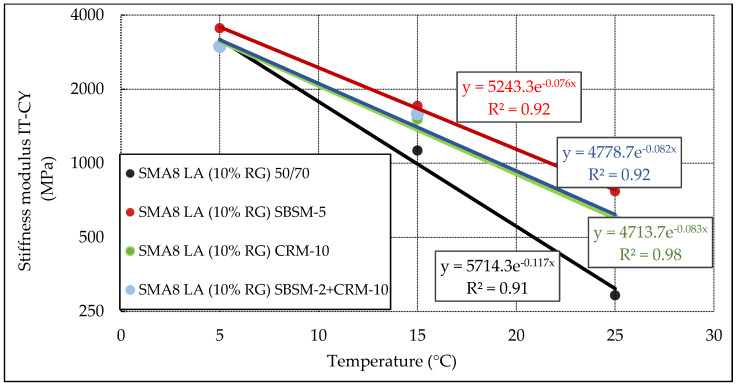
Stiffness modulus IT-CY of SMA8 LA (10% RG) mixtures as a function of temperature, depending on the modified binder.

The analysis of the stiffness modulus of SMA8 LA (10% RG) mixtures in the IT-CY test as a function of temperature is shown in Figure 6. This example confirms that SMA mixtures with modified bitumen have much better resistance to temperature variations (small angle of the trend line in relation to the abscissa axis) compared to the mixture with bitumen 50/70. This means that the higher the resistance to temperature changes, the more the mixture will be resistant to rutting during high temperatures.

### 4.4. Elastic Hysteresis Loops

**Figure 7:**
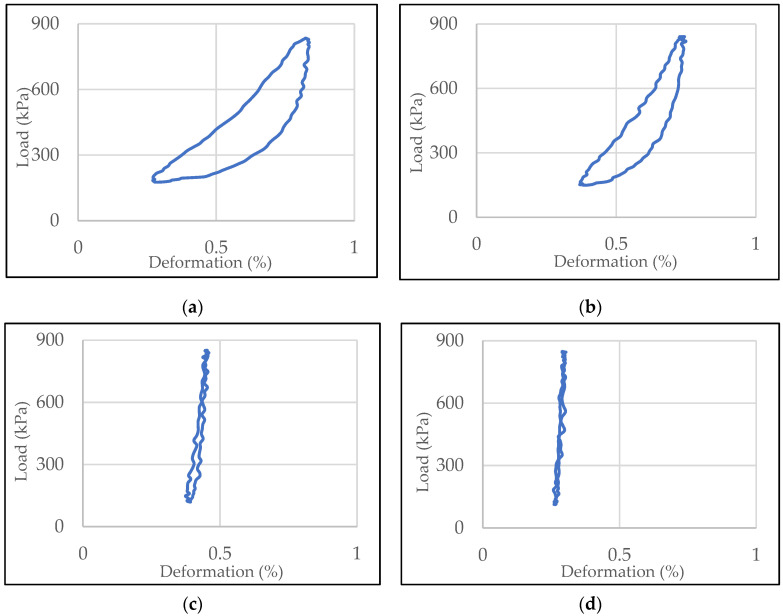
Elastic hysteresis loops of mixtures with modified bitumen CRM-10: (**a**) SMA8 LA (30% RG), (**b**) SMA8 LA (20% RG), (**c**) SMA8 LA (10% RG), (**d**) SMA8 LA.

**Figure 8:**
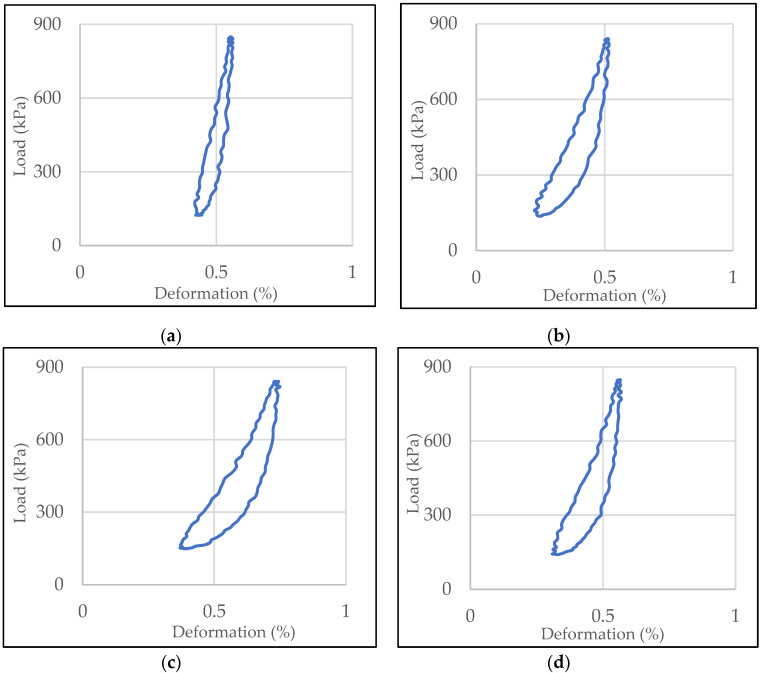
Elastic hysteresis loops of SMA8 LA (20% RG) mixture with: (**a**) bitumen 50/70, (**b**) modified bitumen 50/70 SBSM-5, (**c**) modified bitumen 50/70 CRM-10, (**d**) modified bitumen 50/70 SBSM-2 + CRM-10.

Elastic hysteresis loops of mixtures with SBSM-2 + CRM-10 modified bitumen: SMA8 LA (30% RG), SMA8 LA (20% RG), SMA8 LA (10% RG) and SMA8 LA, are shown in Figure 7 as examples. Elastic hysteresis loops of SMA8 LA mixtures containing 20% rubber granulate with bitumen 50/70 and modified bitumen 50/70 SBSM-5, CRM-10 and SBSM-2 + CRM-10 are presented in Figure 8.

**Figure 9:**
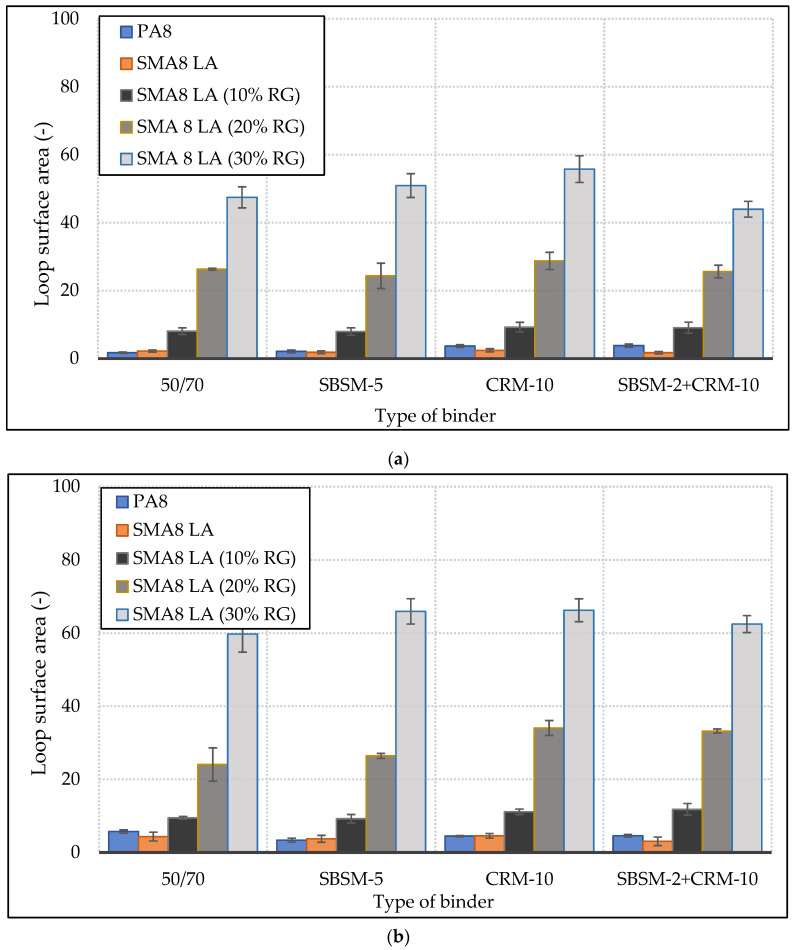

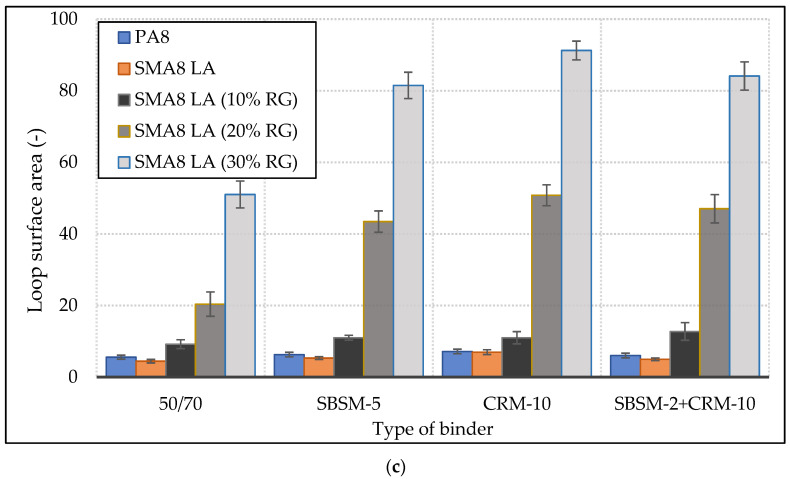
The values of elastic hysteresis loop areas of SMA8 LA and PA8 mixtures at: (**a**) 15°C, (**b**) 25 °C, (**c**) 35 °C.

The areas of the elastic hysteresis loops of SMA8 LA and PA8 mixtures at 15 °C, 25 °C and 35 °C are shown in Figure 9.

The examples of hysteresis loops presented in Figure 7 prove that the addition of rubber granulate to SMA8 LA mixtures has a very significant impact on the mixture elasticity. Increasing the addition of granulate increases the area of the loop. In a situation where loading and relaxation times are equal, the hysteresis loop takes the form of an equilibrium curve (the loop close to the equilibrium curve is shown in Figure 7d. The phenomenon of elastic hysteresis is characteristic for viscoelastic materials, which include asphalt mixtures. It can be predicted that the higher the hysteresis loop area, the more asphalt mixture is able to suppress the sound generated by car tires. The type of binder used has smaller but also significant effect (Figure 8). In the case of SMA8 LA (20% RG) mixtures, the hysteresis loop area for the mixture with bitumen 50/70 is twice smaller compared to mixtures with modified bitumen, for which the order of magnitude of the loop areas is similar. This is particularly evident at the test temperature 35 °C (Figure 9c) for SMA8 LA (20% RG) and SMA8 LA (30% RG) mixtures. The highest values of loop areas were obtained for SMA8 LA (30% RG) mixture with binder modified with crumb rubber (CRM-10)—91.25. This value is 44% higher compared to SMA8 LA (30% RG) mixture with bitumen 50/70 (51.02), then with bitumen SBSM-2 + CRM-10—an increase of almost 40% (84.10) and bitumen SBSM-5—an increase of 37% (81.49).

As the temperature increases, the modified binders give mixtures a higher elasticity compared to the mixtures with the reference bitumen 50/70 (Figure 9). This effect is especially visible at 35 °C (Figure 9c), in which SMA8 LA and PA8 mixtures show lower values of the hysteresis loop area than at 25 °C (except for SMA8 LA). This indicates that bitumen 50/70 loses its elastic properties in the temperature range between 25 °C and 35 °C.

## 5. Conclusions

Based on the water damage resistance test, degradation resistance in the Cantabro abrasion loss test, stiffness modules IT-CY and areas of elastic hysteresis loop performed on stone mastic asphalt SMA8 LA mixtures without and with the addition of rubber granulate and porous asphalt mixture, using SBS copolymer modified binders, crumb rubber or simultaneous modification with SBS copolymer and crumb rubber, the following conclusions were made:

1. The type of binder affects the water damage resistance (ITSR) of SMA8 LA mixtures. Modified binders contribute to the increase of ITSR by about 10% compared to mixtures with bitumen 50/70. The replacement of mineral aggregate by rubber granulate slightly reduces the water damage resistance of SMA8 LA mixtures.
2. The increase in the addition of rubber granulate in SMA8 LA mixtures improves their resistance to degradation in the Cantabro abrasion loss test. The highest degradation resistance achieved SMA8 LA (30% RG) mixtures with copolymer modified bitumen and crumb rubber (SBSM-2 + CRM-10). SMA8 LA mixtures with modified binders are characterized by greater resistance to degradation compared to SMA8 LA with bitumen 50/70. The application of modified binders improves the degradation resistance of SMA LA mixtures by 3–4 times.
3. SMA8 LA, SMA8 LA (10% RG) and SMA8 LA (20% RG) mixtures with modified binders are characterized by higher stiffness modulus values compared to those mixtures with bitumen 50/70. SMA8 LA mixture with modified bitumen with simultaneous copolymer and rubber modification (SBSM-2 + CRM-10) achieved the highest value of stiffness modulus—4082 MPa. This is a 25% increase in modulus value compared to the mixtures with bitumen 50/70. The addition of rubber granulate replacing mineral aggregate reduces the values of stiffness modules of SMA8 LA mixtures.
4. SMA8 LA mixtures with modified bitumen have much higher resistance to temperature changes compared to mixtures with bitumen 50/70. It can be expected that they will be more resistant to rutting in the summer.
5. The addition of rubber granulate to SMA8 LA mixtures has a very significant impact on the elasticity of the mixtures. Increasing the addition of granulate causes an increase in the hysteresis loop area. The highest loop area values were obtained for SMA8 LA (30% RG) mixture with binder modified by rubber (CRM-10). This value is higher by 44% in relation to SMA8 LA (30% RG) with bitumen 50/70, by about 40%- in relation to the mixture with SBSM-2 + CRM-10 and by 37%—in relation to the mixture with SBSM-5.
6. The type of binder significantly affects the values of the hysteresis loop area of SMA8 LA (20% RG) and SMA8 LA (30% RG) mixtures, especially at 35 °C. This means that these mixtures will have a higher capability to suppress vibrations and tire/road noise.
7. As the test temperature increases, the modified binders give the mixtures better elasticity in relation to the mixtures with the reference bitumen 50/70. SMA8 LA mixtures with rubber granulate and bitumen 50/70 show lower hysteresis loop areas at 35 °C compared to 25 °C. This indicates that bitumen 50/70 loses its elastic properties in the temperature range between 25 °C and 35 °C.

## Figures and Tables

**Table 1:** The tested technical properties of modified binders.

Temperature	Units of Measurement	Type of Binder			
		Reference Binder 50/70/After RTFOT	SBSM-5/After RTFOT	CRM-10/After RTFOT	SBSM-2 + CRM-10/After RTFOT
5 °C 15 °C 25 °C	(0.1 mm)	Penetration			
		11.4/8.4	8.7/6.1	8.7/6.8	7.7/5.6
		32.8/20.3	20.6/16.2	19.5/14.1	16.2/13.1
		58.3/44.3	40.2/30.1	40.0/27.8	30.6/24.8
Softening point	(°C)	50.8/56.3	78.6/77.8	60.6/68.2	70.7/77.8
90 °C 110 °C 135 °C	(Pa·s)	Dynamic viscosity			
		11.3/19.3	224.4/258.7	83.6/265.4	292.2/574.1
		2.2/3.6	25.1/27.7	13.4/39.3	43.7/74.9
		0.5/0.7	2.5/3.6	2.1/4.8	5.4/8.8
5 °C 15 °C 25 °C	(J/cm^2^)	Strain energy			
		5.62/6.16	19.40/27.31	5.90/7.25	13.60/17.24
		0.91/2.33	4.38/7.52	2.34/3.57	4.95/7.32
		0.06/0.32	1.39/2.16	0.45/0.88	1.22/2.89
5 °C 15 °C 25 °C	(N)	Maximum tensile strength			
		83.1/118.8	113.6/128.6	88.4/123.3	129.0/147.4
		10.9/18.9	23.9/43.8	24.2/37.0	39.7/45.5
		1.1/3.4	4.8/9.4	4.78.5	10.0/14.2
15 °C 25 °C	(%)	Elastic recovery			
		7.5/15.5	81.9/72.5	65.1/64.4	68.5/70.5
		9.5/16.3	88.7/80.8	71.1/70.2	73.7/73.6

**Table 2:** The physical properties of the aggregate.

Measured Properties	Standard	Specifications	Coarse Aggregate	Fine Aggregate	Limestone Filler
Los Angeles abrasion	EN 1097-2	˂25	17	-	-
Polished stone value	EN 1097-8	>50	54	-	-
Frost resistance in 1% NaCl	EN 1367-6	˂7	4	-	-
Dust content (%)	EN 933-1	coarse ˂ 2	0.7	-	-
		fine ˂ 16	-	2.931	-
Grain density (Mg/m^3^)	EN 1097-6	-	2.890	2.698	-
Grain density (Mg/m^3^)	EN 1097-7	-	-	-	2.671

**Table 3:** The characteristics of tested mixtures.

Measured Properties	Type of Mixture				
	PA8	SMA8 LA	SMA8 LA (10% RG)	SMA8 LA (20% RG)	SMA8 LA (30% RG)
Air void content (%)	23.84	10.56	11.64	11.99	15.05
Binder content (%)	6.3	6.8	8.0	10.0	12.0
Bulk density (Mg/m^3^)	1.954	2.261	2.045	1.819	1.651

**Table 4:** Test results of water damage susceptibility and Cantabro abrasion loss test.

Type of Binder	Type of Mixture									
	PA8		SMA8 LA		SMA8 LA (10% RG)		SMA8 LA (20% RG)		SMA8 LA (30% RG)	
	ITSR	Cant.	ITSR	Cant.	ITSR	Cant.	ITSR	Cant.	ITSR	Cant.
50/70	80.77	13.44	86.12	20,33	84.26	28.47	83.08	17.98	79.75	10.14
SBSM-5	91.44	14.84	95.12	4.40	95.47	7.79	91.24	4.64	89.07	4.91
CRM-10	92.55	14,34	91.97	5.19	88.49	8.53	90.66	8.21	89.52	4.99
SBSM-2+CRM-10	93.57	12.35	92.78	4.73	90.31	8.17	92.10	4.99	87.02	2.22

**Table 5:** Test results of stiffness modulus and area of elastic hysteresis loop for particular types of mixtures depending on the modifier used.

Type of Mixture	Type of Binder	IT-CY (MPa)		Loop Area (-)	
		Temperature (°C)	Average Value	Temperature (°C)	Average Value
PA8	50/70	5	4840	15	1.72
		15	2303	25	5.72
		25	770	35	5.57
	SBSM-5	5	4860	15	2.10
		15	2575	25	3.36
		25	1092	35	6.29
	CRM-10	5	4794	15	3.69
		15	2350	25	4.47
		25	1176	35	7.16
	SBSM-2 + CRM-10	5	3901	15	3.80
		15	2067	25	4.58
		25	1018	35	6.03
SMA8 LA	50/70	5	7894	15	2.22
		15	3207	25	4.34
		25	1181	35	4.44
	SBSM-5	5	6808	15	1.86
		15	3464	25	3.72
		25	1420	35	5.32
	CRM-10	5	6633	15	2.42
		15	3557	25	4.54
		25	1490	35	6.97
	SBSM-2 + CRM-10	5	7526	15	1.71
		15	4082	25	3.05
		25	1754	35	4.99
SMA8 LA (10% RG)	50/70	5	2996	15	8.06
		15	1126	25	9.46
		25	291	35	9.20
	SBSM-5	5	3537	15	7.96
		15	1712	25	9.23
		25	769	35	11.00
	CRM-10	5	2962	15	9.25
		15	1508	25	11.13
		25	566	35	11.00
	SBSM-2 + CRM-10	5	2983	15	9.08
		15	1588	25	11.79
		25	581	35	12.75
SMA8 LA (20% RG)	50/70	5	1196	15	26.32
		15	563	25	24.06
		25	112	35	20.39
	SBSM-5	5	1484	15	24.35
		15	830	25	26.41
		25	119	35	43.45
	CRM-10	5	1235	15	28.75
		15	602	25	34.04
		25	85	35	50.79
	SBSM-2 + CRM-10	5	1202	15	25.61
		15	699	25	33.21
		25	132	35	47.03
SMA8 LA (30% RG)	50/70	5	608	15	47.46
		15	140	25	59.76
		25	60	35	51.02
	SBSM-5	5	691	15	50.92
		15	132	25	65.95
		25	90	35	81.49
	CRM-10	5	485	15	55.75
		15	96	25	66.25
		25	54	35	91.25
	SBSM-2 + CRM-10	5	655	15	43.96
		15	111	25	62.47
		25	66	35	84.10

## References

[1] Sandberg U., Ejsmont J.A. (2002). Tyre/Road Noise. Reference Book.

[2] Praticò F.G., Anfosso-Lédée F. (2012). Trends and issues in mitigating traffic noise through quiet pavements. Procedia-Soc. Behav. Sci..

[3] Gardziejczyk W. (2014). Influence of road pavement macrotexture on tyre/road noise of vehicles. Balt. J. Road Bridge Eng..

[4] Gardziejczyk W. (2016). The effect of time on acoustic durability of low noise pavements—The case studies in Poland. Transp. Res. Part D Transp. Environ..

[5] Mioduszewski P., Gardziejczyk W. (2016). Inhomogeneity of low-noise wearing courses evaluated by tire/road noise measurements using the close-proximity method. Appl. Acoust..

[6] Bendtsen H., Gspan K. (2017). State of the Art in Managing Road Traffic Noise: Noise-Reducing Pavements.

[7] Gӓrtner K. (2014). Lärmreduzierung im Kommunalen BereichDie neuen Merkblätter der FGSV.

[8] Paje S.E., Bueno M., Terán F., Miró R., Pérez-Jiménez F., Martínez A.H. (2010). Acoustic field evaluation of asphalt mixtures with crumb rubber. Appl. Acoust..

[9] Vázquez V.F., Paje S.E. (2016). Study of the road surface properties that control the acoustic performance of a rubberised asphalt mixture. Appl. Acoust..

[10] Paje S.E., Luong J., Vázquez V.F., Bueno M., Miro R. (2013). Road pavement rehabilitation using a binder with a high content of crumb rubber: Influence on noise reduction. Constr. Build. Mater..

[11] Bendtsen H., Andersen B., Kalman B., Cesbron J. The first poroelastic test section in PERSUADE. In Proceeding of the 42nd International Congress and Exposition on Noise Control Engineering.

[12] Ejsmont J.A., Goubert L., Ronowski G., Świeczko-Żurek B. (2016). Ultra low noise poroelastic road surfaces. Coatings.

[13] Jaskula P., Ejsmont J., Stienss M., Ronowski G., Szydlowski C., Swieczko-Zurek B., Rys D. (2020). Initial field validation of poroelastic pavement made with crumb rubber, mineral aggregate and highly polymer-modified bitumen. Materials.

[14] Mioduszewski P., Ejsmont J., Ronowski G., Taryma S. Further development of the poroelastic road surface within the new Polish project SEPOR. Proceedings of the INTER-NOISE and NOISE-CON Congress and Conference.

[15] Liang M., Xin X., Fan W., Wang H., Ren S., Shi J. (2017). Effects of polymerized sulfur on rheological properties, morphology and stability of SBS modified asphalt. Constr. Build. Mater..

[16] Pakholak R., Plewa A., Hatalski R. (2020). Evaluation of selected technical properties of bitumen binders modified with SBS copolymer and crumb rubber. Struct. Environ..

[17] Mazzotta F., Tataranni P., Simone A., Fornai D., Airey G., Sangiorgi C. (2020). Multi-scale rheo-mechanical study of sma mixtures containing fine crumb rubber in a new dry-hybrid technology. Appl. Sci..

[18] Li L., Guo Z., Ran L., Zhang J. (2020). Study on low-temperature cracking performance of asphalt under heat and light together conditions. Materials.

[19] Pszczoła M., Ryś D., Jaskuła P. (2017). Analysis of climatic zones in Poland with regard to asphalt performance grading. Roads Bridges-Drog. i Mosty.

[20] Mackiewicz P., Szydło A. (2019). Viscoelastic parameters of asphalt mixtures identified in static and dynamic tests. Materials.

[21] Wahengbam R.D., Rajbongshi P. (2015). An approach for dynamic stiffness evaluation in asphalt concrete. Constr. Build. Mater..

[22] Tiraturyan A.N., Uglova E.V., Lyapin A.A. (2017). Studying the energy distribution of the dynamic influences of road transport on the layers of nonrigid pavements. PNRPU Mech. Bull..

[23] Shirini B., Imaninasab R. (2016). Performance evaluation of rubberized and SBS modified porous asphalt mixtures. Constr. Build. Mater..

[24] Mazurek G., Iwański M. (2018). Multidimensional analysis of the effects of waste materials on physical and mechanical properties of recycled mixtures with foamed bitumen. Appl. Sci..

[25] Iwanski M., Chomicz-Kowalska A., Maciejewski K. (2019). The influence of hydrated lime on IT-CY stiffness modulus of foam-based asphalt concrete compacted at 95 °C. Mater. Sci. Eng..

[26] European Standard: EN 12607-1:2014. Bitumen and Bituminous Binders—Determination of the Resistance to Hardening Under Influence of Heat and Air-Part 1: RTFOT Method. https://www.sis.se/api/document/preview/104363/.

[27] European Standard: EN 12697-30:2019. Bituminous Mixtures—Test Methods—Part 30: Specimen Preparation by Impact Compactor. https://www.sis.se/api/document/preview/80009154/.

[28] Poland Technical Requirements: TR-2 2014. Asphalt Pavements on National Roads—Asphalt Mixtures—Part 2. https://www.gddkia.gov.pl/frontend/web/userfiles/articles/d/dokumenty-techniczne_8162/Dokumenty%20techniczne/WT2%20cz1.pdf.

[29] ASTM D6931-17 (2017). Standard Test Method for Indirect Tensile (IDT) Strength of Asphalt Mixtures.

[30] European Standard: EN 12697-17:2017. Bituminous Mixtures—Test Methods—Part 17: Particle Loss of Porous Asphalt Specimens. https://www.sis.se/api/document/preview/8025276/.

[31] Sangiorgi C., Tataranni P., Simone A., Vignali V., Lantieri C., Dondi G. (2018). Stone mastic asphalt (SMA) with crumb rubber according to a new dry-hybrid technology: A laboratory and trial field evaluation. Constr. Build. Mater..

[32] European Standard: EN 12697-26:2018. Bituminous Mixtures—Test Methods—Part 26: Stiffness. https://www.sis.se/api/document/preview/80004874/.

[33] European Standard: EN 12697-25: 2016. Bituminous Mixtures—Test methods—Part 25: Cyclic Compression Test. https://www.sis.se/api/document/preview/8021917/.

[34] Hysteresis Phenomenon. http://www.koros-plast.ru/stroenie-polimerov/yavlenie-gisterezisa.html.

